# Family physicians enhance end-of-life care: evaluation of a new continuing medical education learning module in British Columbia

**DOI:** 10.1186/s12909-015-0392-4

**Published:** 2015-07-24

**Authors:** Helena Kadlec, Marcus J. Hollander, Catherine Clelland, Liza Kallstrom, Marcus Hollander

**Affiliations:** 1 Hollander Analytical Services Ltd, 300-895 Fort Street, Victoria, BC Canada; 2 Practice Support Program, Doctors of BC, 115 - 1665 W. Broadway, Vancouver, BC V6J 5A4 Canada

**Keywords:** End-of-life care, Primary care, Continuing education, Practice change, Evaluation

## Abstract

**Background:**

The Practice Support Program (PSP) is an innovative peer-to-peer continuing medical education (CME) program that offers full-service family physicians/general practitioners (GPs) in British Columbia (BC), Canada, post-graduate training on a variety of topics. We present the evaluation findings from the PSP learning module on enhancing end-of-life (EOL) care within primary care.

**Methods:**

Pen-and-paper surveys were administered to participants three times: at the beginning of the first training session (*n* = 608; 69.6 % response rate), at training completion (*n* = 381, 55.6 % response rate), and via a mail-out survey at 3-6 months following training completion (*n* = 109, 24.8 % response rate). Surveys asked GPs about current EOL-related practices and confidence in EOL-related skills. At end of training, respondents also provided ratings of satisfaction and perceptions of the module’s impact on their practice and their EOL patients.

**Results:**

Satisfaction and impact were rated very highly by over 90 % of the GP respondents. Module participation increased the GPs’ confidence on EOL-related communication and collaboration skills: e.g., initiating conversations about EOL care, developing an action plan for EOL care, communicating the patient’s needs and wishes to other care providers, participating in collaborative care with home and community care nurses, and accessing and referring patients to EOL specialists in the community. Increased confidence was maintained at 3-6 months following completion of training.

**Conclusions:**

The EOL learning module offered by the PSP to family physicians in BC is a successful and impactful CME accredited training module for enhancing end-of-life care in primary care settings.

## Background

A novel continuing medical education (CME) program, the Practice Support Program (PSP) previously described by MacCarthy et al. [1, 2], continues to be offered to family physicians/general practitioners (GPs) and their staff in the province of British Columbia (BC), Canada. The PSP involves peer-to-peer training on a number of clinical and office management topics that are offered in learning modules for CME credit (see http://www.gpscbc.ca/practice-support-program). MacCarthy et al. [1] reported the results from the initial four PSP learning modules on chronic disease management, group medical visits, patient self-management, and advanced access/office efficiency, and MacCarthy et al. [2] reported on the adult mental health module. Here we report the evaluation results from the PSP learning module that offers CME accredited training to GPs to enhance care for their patients who require end-of-life (EOL) care and their families. We emphasize that this learning module was on end-of-life care and encompassed the palliative approach to care.

GPs often have limited formal education in end-of-life care, and their knowledge, skills and confidence in providing this type of care varies (e.g., [3]). For example, a survey of family physicians in Germany found that 53 % of GPs reported that they gained their first experience in palliative medicine as junior hospital doctors and another 26 % only after starting their private practice [4]. Although the case load for patients requiring end-of-life or palliative care is typically low, it is consistent, and a GP can expect to care for between three and six terminally ill or dying patients per year ([5], see also [3]).

In terms of availability of CME programs for EOL care for primary care physicians, a 2006 systematic review of educational opportunities identified 18 articles published between 1966 and 2005 [5]. The available CME programs for palliative care (which these authors defined as encompassing terminal care) included role model training, small group discussions and distribution of guidelines. The formats of the various programs included GP-facilitated workshops, long-term (e.g., 3-year) mentorships, year-long web-based courses, and monthly multi-professional study days. Most of the programs employed multifaceted educational approaches and were based on adult educational principles. Positive outcomes of the reviewed studies indicated: improvements in GPs’ knowledge and attitude scores with regard to pain and symptom management; greater understanding of other team members’ work stemming from multi-professional participation opportunities; and the participating GPs’ overall satisfaction with the CME program. On the other hand, when assessed in the evaluation, there were small or no changes noted in communication with patients and more objective measures such as opioid prescriptions or pain assessment [5]. A more recent review of CME offerings for palliative care in primary practice available in Belgium indicated large content gaps, under-subscription to the “labyrinth” of courses, and a general lack of evaluation of the impact of the CME courses on clinical practice [6].

Within this global context, the PSP is similar to other CME offerings in terms of the content delivery format, but offers two somewhat unique features. Similarly to the other CME courses, the PSP modules follow the established adult education principles that are most likely to lead to behaviour change in GPs’ practices, by offering multifaceted, multi-professional and interactive learning opportunities. Furthermore, the content of each learning module is developed by faculty experts in the content area who themselves train a cohort of GP “champions” or facilitators from across the province. The GP facilitators then deliver the modules to their colleagues in their respective regions, with additional administrative support from dedicated PSP regional staff members and where available with team support from other health care providers. For the EOL module, these include home and community care (HCC) nurses and/or palliative care (PC) nurses. The GP participants can also invite a medical office assistant (MOA) or other key staff person to participate in the module with them, although not every GP does. The typical delivery format includes three face-to-face learning sessions (each about 4 h long), interspersed with 2-3 month long action periods where each module participant practices the new learnings in his/her own office setting with the support of the GP champion and the PSP regional team member.

**Fig. 1:**
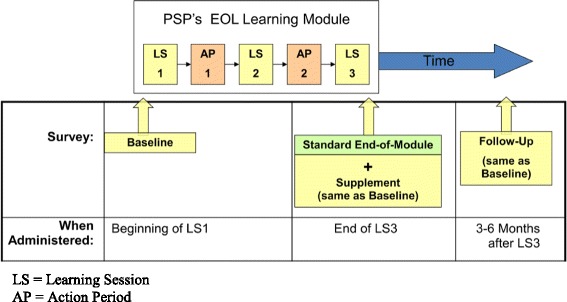
Overview of the EOL Evaluation Surveys

Compared to most of the other CME programs reviewed, the PSP is much larger in scale. It is available to all GPs and their staff across the whole province of British Columbia. To date, 2797 (78 %) of all GPs in BC have participated in the PSP (across all modules), and during the period of the evaluation of the EOL module (from implementation in September 2011 to end of June 2013), 873 GPs had participated in the EOL module. The other distinguishing feature of the PSP is that the program evaluation was conducted by an independent research team. The evaluation design could not be a randomized control trial due to the nature of the roll-out of the learning modules across the province, but the participants were surveyed before training, at the end of training, and at 3-6 months following completion of training to assess the retention or sustainability of the new knowledge and skills (see Fig. 1).

In this report, we present the findings from the evaluation surveys of the GPs who participated in the EOL learning module. We report on two sets of outcomes for the specific learning objectives of the EOL module’s faculty and content developers: (1) the GPs’ satisfaction with the module, including their perceptions of whether the module had met its objectives and its impact on their practice and their end-of-life patients, and (2) a number of EOL-specific outcomes and their changes across time, including GPs’ self-rated knowledge, communication skills, collaborations with other care providers, and conducting home visits.

## Methods

### Participants

During the evaluation of the EOL Learning Module, between September 2011 and June 2013, a total of 608 GPs completed the EOL baseline survey, 381 completed the end-of-module survey and 109 completed the 3-6 month follow-up survey. The response rates were: 69.6 % on the Baseline survey (out 873 GPs); 55.6 % on the End of Module survey (of the 685 GPs who had completed the EOL module by the end of the evaluation period; this included GPs who did not complete the baseline survey); and 24.8 % on the mail-out 3-6 month Follow-up survey (of the 440 GPs who had reached the 3-6 month post-module evaluation date). Table 1 shows the description of the GP respondents on each of the three surveys.

The respondents on the end-of-module survey were also divided into 6-month intervals, to identify three types of “adopters”, with “early” adopters (155 GPs) being the first set of cohorts who completed the training (and the end-of-module survey) in the first wave of participation, between January and June 2012. The “middle” adopters were 90 GPs who completed their training (and end-of-module survey) between July and December 2012. And the” late” adopters were 104 GPs who completed their training in the last 6 months of the evaluation period. (Note that 30 GPs (7.9 %) did not provide the date of their completion, so these respondents could not be categorized for these analyses.)

### Survey questionnaires

Two types of surveys were employed in this evaluation. One survey assessed the participants’ self-reported end-of-life practices at three points in time: prior to training (at the beginning of the first learning session, to establish the baseline); at the end of training (to assess the impact of the new learning); and at 3-6 months following module completion (to assess the retention/sustainability of the new practices). The second survey, called the end-of-module survey, was administered with the EOL-Practices survey at the end of the training period, and asked participants to rate their perceptions of the EOL Learning Module (see Fig. 1).

Seven socio-demographic variables were asked on all surveys: sex; age group (in decade categories); years worked in family practice; whether the participant worked full- or part-time (referred to as work pattern below); the type of practice – solo (one GP), two-physician, small group practice (3 to 4 GPs), large group practice (5 or more GPs), walk-in clinics and specialized services/other; size of practice – fewer than 1000 individual patients seen per year, 1000 to 1999 patients, 2000 to 2999 patients, and 3000 or more patients; and size of community – metropolitan, urban/suburban, town, or rural/remote, as defined by Olatunde et al. [7]. These seven, along with the type of adopter, also served as independent variables in several analyses that follow.

The EOL-Practices survey asked GPs about EOL-related care highlighted by the learning objectives identified by the faculty and PSP content developers. Specifically, these were questions about their current EOL-related practices (i.e., having a registry, action plans, guidelines, collaborating with HCC, conducting  home visits) and their self-rated confidence on EOL-related skills (e.g., identifying patients who would benefit from a palliative approach, initiating a conversation about end-of-life care with a patient, developing an action plan for patients who require end-of-life care, guiding patients with regards to their goals of care at the end of life, supporting patients during the terminal phase of their illness, and supporting the patient’s family during grief and bereavement). Confidence ratings were solicited on a 4-point response scale ranging from 1 = very confident to 4 = not at all confident.^1^

The end-of-module survey contained three sets of questions in addition to the demographic questions. It asked respondents to rate: (1) their satisfaction with the learning module; i.e., with the learning sessions, the action periods, and the goals and measures used to assess practice changes (22 items in total) that formed the Satisfaction Scale; (2) their perceptions of the impact of the EOL module on their practice and their patients in general (20 items) that formed the Perceived Impact Scale; and (3) key objectives with regard to EOL-related care (13 items) that formed the EOL-Objectives Scale. Responses to these questions were scored on a 5-point agree-disagree scale, ranging from strongly disagree (1) to neither agree nor disagree (3) to strongly agree (5).

### Data collection and analyses

Data collection of the EOL-Practices baseline and end-of-module surveys was conducted at the learning sessions. The baseline survey was completed by the participants at the beginning of the first learning session. The end-of-module survey was completed at the end of the third learning session. There were strong expectations that the surveys would be completed at the sessions and the GPs were remunerated as part of their attendance at the learning sessions (there was dedicated time at the session for its completion). The 3-6 month follow-up survey of participants who completed the EOL module was conducted by mail, and GPs were remunerated $40 CAD for returning the completed survey.

For statistical analyses, we examined differences in proportions using *χ*^2^ tests and differences in group means using one-way or two-way analyses of variance (ANOVAs). To control for Type I error rates (family-wise), multivariate ANOVAs (MANOVAs) were conducted on the subsets of survey items, and post-hoc tests used the Bonferroni adjustment.

As per the Canadian requirements for conducting research with human participants (see “The Tri-Council Policy Statement: Ethical Conduct for Research Involving Humans” document, pp.20 at http://www.pre.ethics.gc.ca/pdf/eng/tcps2/TCPS_2_FINAL_Web.pdf), a formal ethics review for conducting evaluations of quality improvement initiatives is not required in Canada. However, this evaluation conformed to all standard practices of ethical research, including the obtaining of informed consent from all participants, confidentiality of data, and anonymity of all survey respondents.

## Results and discussion

We report the results in three sections. First, we present the module participants’ evaluation of the learning module from the end-of-module survey, namely their ratings of satisfaction and the impact that they perceived the EOL module had had on their practices and their patients. In this section, we report the GPs’ responses as a function of the seven socio-demographic variables and type of adopter.^2^ In the second section, we present the changes in the GPs’ self-reported practices in regards to providing EOL care to their patients and their families and caregivers, and in the third section, we report the GPs’ changes in their self-rated confidence in their EOL related knowledge and skills. In the second and third sections, we also report the GPs’ responses as a function of when they participated in the learning module (by comparing the responses across adopter groups) and the seven socio-demographic variables.

### Satisfaction, perceived impact and EOL-objectives ratings: end-of-module survey results

**Fig. 2:**
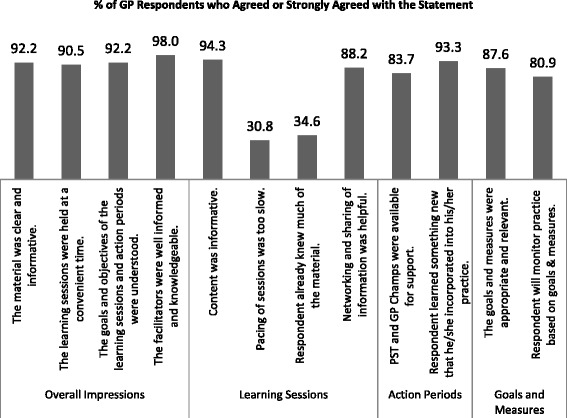
Ratings of Satisfaction with the EOL Module

**Fig. 3:**
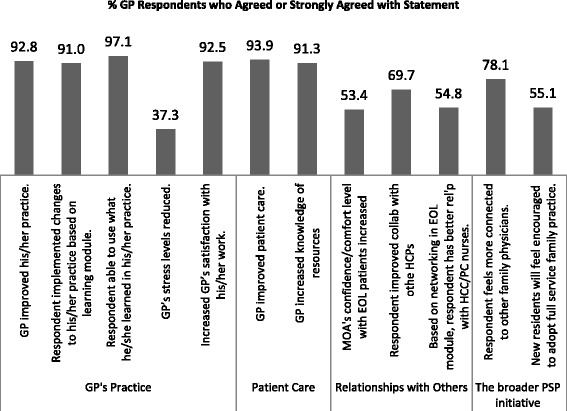
GPs’ Ratings of Impact of the EOL Module on GPs’ General Practices, Patients and Relationships with other Providers

**Fig. 4:**
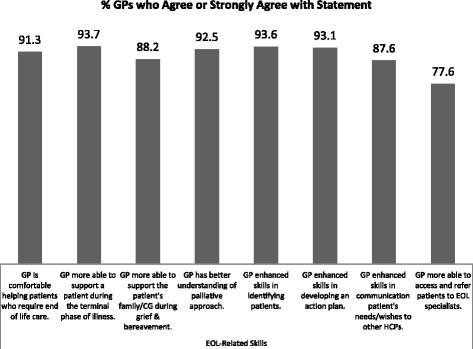
GPs’ Ratings of Impact on their EOL Understanding and Related Practices

Satisfaction with the learning module – i.e., with the learning sessions, action periods, and goals and measures – was rated high by large majorities of the GP respondents. Figure 2 shows the percentages of GP respondents who agreed or strongly agreed with some key satisfaction items on the survey. (In Figs. 2, 3 and 4, we collapsed the two response categories of “strongly agree” and “agree” to simplify the presentation of the results.) To examine whether the satisfaction ratings with the module differed by the respondents’ socio-demographic variables and type of adopter, the ratings were summed to form Satisfaction Scale scores (Cronbach’s alpha = .8864), and one-way ANOVAs were conducted on the group mean ratings. The Satisfaction Scale scores did not differ across respondents’ gender, age group, work pattern, practice size, type of community, years in practice,^3^ or type of adopter (all *p*s > .0715). There was, however, a statistically significant difference in Satisfaction Scale scores between GPs who work in different types of practices (F(3,336) = 3.86, *p* = .0097, MSE = 300.68, η^2^ = .0333). Post-hoc tests using Bonferroni correction for Type I error rates showed that the highest satisfaction ratings were received from physicians in two-GP practices followed by physicians who worked in large (5 or more GP practices). Satisfaction ratings from solo GPs and those in small group (3 to 4 GPs) practices were comparatively the lowest among these groups, although, it must be stressed, still very high.

For the Perceived Impact Scale scores (Cronbach’s alpha = .9134), the only statistically significant differences in ratings were between men and women, F(1,206) = 7.15, p = .0081, MSE = 90.01, η^2^ = .033. Women GPs rated the general impact of the EOL module on their practice and their patients higher than the men, and although it was statistically significant, the size of the effect was negligible (an average of 0.18 points per item on the 4-point rating scale). None of the other socio-demographic variables or type of adopter (i.e., wave of participation) were associated with the Perceive Impact ratings (all *p*s > .0908). Ratings from all GP respondents on selected items of this scale are shown in Fig. 3.

The EOL-Objectives Scale scores (Cronbach’s alpha = .8852) were related to two socio-demographic variables. First, the size of the GP’s practice influenced their ratings (F(4, 348) = 3.17, *p* = .0140, MSE = 36.43, η^2^ = .0352); and post-hoc tests revealed that GPs who did not know how many individual patients their practice served rated the impact of the module higher than GPs in other groups. Second, a negative correlation coefficient was found between years in practice and the scale scores (Pearson Product r = -.1330, N = 374, *p* = .0110) which indicated that GPs who had practiced family medicine longer tended to rate the perceived impact lower. This is not surprising, as more experienced GPs would have had more practice with patients who required EOL care during their longer careers. Type of adopter was not related to the EOL-Objectives Scale scores. GPs’ ratings on selected items of this scale are shown in Fig. 4.

### EOL-related practices: changes from baseline to end-of-module to 3-6 month follow-up

As shown in Table 2, attending the EOL learning module changed the GPs’ practice to a significant and strong degree. The GPs increased all EOL-related activities. Post-hoc tests, with Bonferroni correction, indicated that:

**Table 1:** Socio-demographic description of GP respondents on the three evaluation surveys

		Survey		
		Baseline	End of Module	Follow-Up
	Number of Surveys Completed (Total N)	608	381	109
	Number of Surveys Administered	873	685	440
	Survey Response Rate	69.6 %	55.6 %	24.8 %
Gender	Males	307 (50.5 %)	190 (49.9 %)	56 (51.4 %)
	Female	293 (48.2 %)	186 (48.8 %)	52 (47.7 %)
	No response	8 (1.3 %)	5 (1.3 %)	1 (0.9 %)
Age Group	29 and younger	10 (1.6 %)	2 (0.5 %)	0
	30-39	114 (18.8 %)	76 (19.9 %)	14 (12.8 %)
	40-49	174 (28.6 %)	108 (28.4 %)	37 (33.9 %)
	50-59	204 (33.6 %)	132 (34.6 %)	45 (41.3 %)
	60-69	92 (15.1 %)	53 (13.9 %)	11 (10.1 %)
	70 and older	12 (2.0 %)	3 (0.8 %)	1 (0.9 %)
	No response	2 (0.3 %)	7 (1.8 %)	1 (0.9 %)
Years in Practice	Mean (SD)	20.6 (11.4)	19.4 (10.7)	21.2 (9.1)
Pattern of Work	Part-time	131 (21.6 %)	83 (21.8 %)	28 (25.7 %)
	Full-time	461 (75.8 %)	283 (74.3 %)	80 (73.4 %)
	Other/No response	16 (2.6 %)	15 (3.9 %)	1 (0.9 %)
Nature of Practice (Where work most often)	Solo GP	79 (13.0 %)	40 (10.5 %)	10 (9.2 %)
	Two GPs	89 (14.6 %)	56 (14.7 %)	15 (13.8 %)
	3-4 GPs	161 (26.5 %)	120 (31.5 %)	34 (31.2 %)
	5 or more GPs	232 (38.2 %)	141 (37.0 %)	43 (39.5 %)
	Specialized Services/Other	39 (6.4 %)	11 (2.9 %)	6 (5.5 %)
	No response	1 (0.2 %)	13 (3.4 %)	1 (0.9 %)
Size of Practice	Fewer than 1000 patients	113 (18.6 %)	46 (12.1 %)	16 (14.7 %)
	1000-1999 patients	250 (41.1 %)	150 (39.4 %)	43 (39.5 %)
	2000 to 2999 patients	93 (15.3 %)	75 (19.7 %)	25 (22.9 %)
	3000 or more patients	66 (10.9 %)	50 (13.1 %)	14 (12.8 %)
	Primarily a walk in clinic	7 (1.2 %)	3 (0.8 %)	2 (1.8 %)
	Don’t know/no response	79 (13.0 %)	57 (13.2 %)	9 (8.3 %)
Type of Community	Metropolitan	172 (28.3 %)	156 (40.9 %)	37 (33.9 %)
	Urban/Suburban	125 (20.6 %)	138 (36.2 %)	43 (39.5 %)
	Town	0 (0 %)	65 (17.1 %)	23 (21.1 %)
	Rural/Remote	22 (14.5 %)	20 (5.2 %)	5 (4.6 %)
	More than one region	4 (0.7 %)	1 (0.3 %)	0
	No response	197 (32.4 %)	1 (0.3 %)	1 (0.9 %)

**Table 2:** Respondents’ EOL-related practices across time

	Baseline (*N* = 554 to 595)	End-of-Module (*N* = 347 to 372	Follow-Up (*N* = 104 to 106)	Inferential test statistic; *p*-value
Currently have a registry for patients requiring end of life care: % GPs responding yes	7.9 %	65.9 %	52.4 %	*χ*^2^(2) = 372.92; *p* < .0001
Currently have an action plan for patients who require end of life care: % GPs responding yes	28.3 %	68.9 %	83.7 %	*χ*^2^(2) = 206.01; *p* < .0001
Participate in collaborative care with Home and Community Care and/or Palliative Care providers: Mean (SD) Rating^a^	2.33 (1.06)	2.22 (0.92)	1.66 (0.72)	F(2,1069) = 21.75; *p* < .0001
Generally follow the most recent clinical guidelines for palliative care: % GPs responding yes	61.7 %	88.8 %	94.3 %	*χ*^2^(2) = 106.58; *p* < .0001
Currently conduct home visits for patients requiring end-of-life care: Mean (SD) Rating^a^	2.85 (1.23)	2.61 (1.16)	2.58 (1.16)	F(2,1062) = 5.49; *p* = .0043

^a^ The response scale for this item ranged from 1 = always to 5 = never, thus lower numbers indicate a more frequent occurrence of the practice

- The percentage of GPs who developed a registry for patients requiring EOL care rose dramatically during the course of the EOL module (from 7.9 % to 65.9 %). However, while a majority (52.4 %) of GPs continued to use their registry at 3-6 month following completion of the module, the 13.5 % drop was statistically significant (*p* = .004).
- The percentage of GPs who developed an action plan for their patients rose significantly during the course of the module and continued to rise following the completion of the module.
- Participation in collaborative care with HCC and/or PC providers did not see a rise in frequency during the course of the EOL module but did become more frequent during the follow-up period (*p* < .001 from baseline and end-of-module).
- The percentage of GPs who indicated that they follow clinical guidelines for palliative care increased from baseline to end of module and follow-up, although the additional increase from end-of-module (88.8 %) to follow-up (94.3 %) was not statistically significant (*p* = .671).
- For home visits, the increase in frequency from baseline to end-of-module was statistically significant (*p* = .008), but no further increase was noted during the follow-up period.

These findings indicate that the EOL module had a strong impact on the GPs’ self-reported changes in their practices vis-à-vis patients requiring end of life care. It is also noteworthy that many behaviour changes were sustained for at least 3 to 6 months following the completion of the module, suggesting that the new learnings were well incorporated into the GPs’ care practices.

To examine whether any of the socio-demographic variables were related to changes in these practices, two-way MANOVAs were conducted, with type of survey (baseline, end-of-module, follow-up) and each demographic variable as the two independent variables.^4^ Responses on these five EOL activities served as the dependent variables. As noted with the univariate results reported above, the main effect of type of survey, i.e., changes across time, was statistically significant in all two-way MANOVAs (all p-values < .0001). The following results were found with regard to the socio-demographic variables.

There was no effect of gender (interaction with survey type, *p* = .7974; main effect *p* = .1161) nor pattern of work (part- versus full-time) (interaction with survey type *p* = .9330; main effect *p* = .0618). There were, however, main effects of age group (recoded into 4 groups) (Wilks’ Lambda = 0.9672, F(15, 2581.5) = 2.09, *p* = .0082), type of practice (Wilks’ Lambda = 0.9190, F(30, 3694.0) = 2.63, *p* < .0001), and type of community (Wilks’ Lambda = 0.9401, F(20, 2554.8) = 2.40, *p* = .0005). (None of the two-way interactions with survey type were statistically significant in these MANOVAs, all *p*s > .2532.)

Post-hoc analyses revealed that the GP age groups differed with regard to collaborating with HCC and/or PC nurses/providers (*p* < .0001), following guidelines (*p* = .0295) and conducting home visits (*p* < .0001), but not in having registries and action plans for patients requiring EOL care. Generally, older GPs tended to conduct the EOL-related activities more frequently. Specifically, the youngest age group (20-39 year old GPs) reported the least frequent collaborations with HCC/PC providers, followed by the middle age group (40-49 year old GPs), and the two oldest age groups (50-59 and 60 or older) reporting more frequent collaborations. With regard to conducting home visits, the youngest age group (20-39 year old GPs) conducted home visits less frequently that the three older age groups, which did not differ from each other. And with regard to following palliative care guidelines, the only statistically significant difference was between the youngest and oldest age groups (*p* = .037). As before, these findings are not surprising: older GPs have more experience with these aspects of care provision. It is interesting, however, that in terms of the registries and developing action plans there were no age group differences suggesting all GPs were at a similar stage of using these tools in their practices.

Post hoc analyses of the multivariate main effect of type of practice indicated that the only group difference was in conducting home visits. GPs in 2-physician practices reported that they conduct home visits more frequently than physicians in large group (5+ physicians) practices, whereas GPs who work primarily in walk-in clinics and in specialized services report that they conduct home visits least frequently (average rating was between “occasionally” and “rarely”). No group differences were found in having registries, formulating action plans, collaborating with HCC/PC providers, and following palliative care guidelines.

Differences between types of communities were found on the two practices of collaborating with HCC/PC nurses (*p* < .0001) and conducting home visits (*p* < .0001). GPs in metropolitan areas reported that they collaborated less frequently with HCC/PC providers and made less frequent home visits when compared to GPs from urban/suburban, town and rural/remote communities (*p* < .0001 to *p* = .022). Furthermore, GPs from urban/suburban areas also reported less frequent home visits than GPs from townships and rural/remote areas (*p* = .002 and *p* = .019, respectively).

### Confidence with EOL-related skills and knowledge

**Fig. 5:**
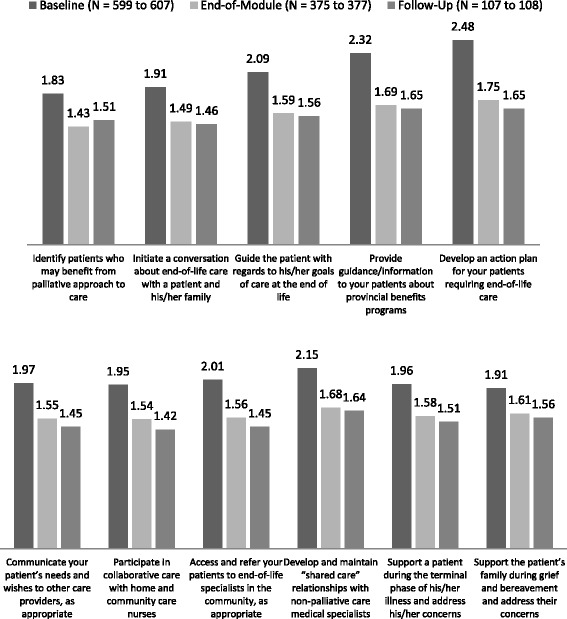
GPs’ Average Confidence Ratings on EOL-Related Skills Across Time (Smaller values indicate higher confidence: 4-point response scale ranged from 1 “very confident” to 4 “not at all confident”) *p* < .0001 for all differences between baseline and end-of-module means

The GPs were asked to rate their confidence on 11 skills/abilities and three general knowledge areas related to providing end-of-life care. Changes in their confidence ratings on the EOL-related skills, from baseline to follow-up, are shown in Fig. 5. A MANOVA on the 11 skills showed statistically significant differences across time (Wilks’ Lambda = .7483, F(22, 2058) = 14.59, *p* < .0001). Post-hoc analyses indicated that each of the 11 skills showed a statistically significant change over time (univariate ANOVA F-values ranged from 29.28 to 156.94 and all *p*s < .0001). For all 11 skills, the increase in confidence occurred between baseline and end-of-module, and although for most of the skills there was a further, albeit slight, increase in confidence from module completion to the follow-up, none of these were statistically significant (all *p*s > .3310).

To examine whether any of the socio-demographic variables were related to the GPs’ changes in confidence in providing EOL care, two-way MANOVAs were conducted, with survey (baseline, end-of-module, follow-up) and each demographic variable serving as the independent variables, and the confidence ratings on the 11 EOL-related skills serving as the dependent variables. In all MANOVA analyses, the main effect of survey type (i.e., differences in ratings across time) was statistically significant (*p* < .0001), none of the demographic variables interacted with the survey type (all p-values for the interactions were greater than .2650). Three demographic variables showed a main effect on the confidence ratings: Gender (Wilks’ Lambda = .9690, F(11,1013) = 2.95, *p* = .0007), age group (Wilks’ Lambda = .9515, F(33,2979.3) = 1.54, *p* = .0261), and work pattern (Wilks’ Lambda = .9778, F(11,996) = 2.06, *p* = .0211). Type of practice and type of community showed no relationship with the GPs’ confidence ratings (p = .9302 and .5693, respectively).

Post-hoc, univariate analyses indicated that: Although the mean differences were very small (ranging from .093 to .161 on the 5-point rating scale), male GPs rated their confidence statistically higher on six of the 11 skills (identifying EOL patients, initiating EOL conversations, guiding patients on goals, developing action plans, referring patients to specialists, and supporting the patient during the terminal phase of the illness). Confidence ratings differed across age groups on all 11 skills, with the predictable pattern of older GPs rating their confidence higher, consistently across all four age groups and on each skill (all p-values in the one-way ANOVAs were ≤ .0009). And while work pattern had a significant main effect in the MANOVA, only one of the 11 skills showed a marginally statistically significant difference in the post-hoc univariate analyses, and that was that full-time GPs were slightly more confident in supporting a patient during the terminal stage of their illness than part-time GPs (*p* = .0694).

## Conclusions

The PSP module was received very well by the participating GPs. GPs rated their satisfaction with the module highly, indicated that the learning objectives were met and the impact on both their practices and their patients was positive. In addition, the GPs’ ratings from the follow-up survey indicated that the changes that they had made in their practices with regard to providing EOL care to their patients were sustained, and in some cases improved, in the 3 to 6 months following module completion, at least for those GPs who completed the follow-up survey.

Practices related to providing EOL care as well as the GPs’ confidence in providing those services to their patients increased, in many cases dramatically, over the course of the learning module and in the 3 to 6 months following the completion of the module. This is very encouraging, and indicates that the new approaches and skills were implemented and well integrated into the participants’ practices.

A positive outcome in this evaluation was the GPs’ increased confidence in their skills for communicating with their patients and patients’ families regarding end-of-life issues. After completion of the module and maintained into the 3 to 6 month post-module period, the GPs felt considerably more confident in initiating conversations about end-of-life care with their patients and communicating the patients’ needs and wishes to other care providers as appropriate. They were more confident guiding their patients with regards to the patient’s goals of care, developing action plans, and supporting the patient during the terminal phase of the illness as well as the patient’s family during grief and bereavement.

Although participating in the EOL module also increased the GPs’ self-reported confidence in participating in collaborations, making referrals, and “sharing care” for a patient within a care team setting, the frequency of collaborations with other providers, namely home and community care and palliative care nurses, were not as strongly impacted by the EOL module. However, some progress was seen, particularly in the frequency of participation with HCC and PC providers since completion of the module. This is one area that could be further strengthened within the delivery of this learning module, or perhaps it simply takes more time to establish and grow those relationships within the GP’s community. Mitchell [3] found that GP comfort working with specialist teams increases with exposure to this form of patient management, and formal arrangements that engage GPs within specialist teams have been shown to improve: functional outcomes, patient satisfaction, effective use of resources, understanding of the roles and potential that other team members contribute to patient care, and generally improve effective physician behaviour in other areas of medicine as well.

There are several limitations to our study. One key one was lack of a control group, but it was not possible to include a control group given the nature of the PSP program and its rollout of the learning modules across the province. Second is the self-selection of the GP respondents. Our response rates on the baseline and end-of-module surveys were reasonably high, but for the follow-up survey, which was a mail-out survey, the response rate was in the typical 25 % range. It is also likely that only those GPs who maintained the new EOL practices were more likely to respond to the survey, and may thus be a non-representative sample of those who completed the EOL module. That said, however, even if a hundred GPs across the province indicated a positive impact on their practices and patients at 3 to 6 month following the completion of the learning module, that is itself a positive outcome. A third limitation is the lack of patient and family/caregiver perspective on the GPs’ new skills. We considered surveying the patients and/or key family members, but decided not to: we felt that surveying terminal patients and their families during such an emotional and difficult time was fraught with too many issues and would make both data collection and data interpretation difficult. A fourth limitation is that all the findings reported here are based on the GPs’ perceptions and self-ratings, and we cannot assess whether in fact any changes in practice have been implemented.

The strengths of our study are the large sample sizes of the three surveys, and the relatively high response rates of the participating GPs. This gives us confidence that the findings we reported here represent the GPs’ perceptions that the EOL learning module offered by the PSP for all family physicians within the province is an excellent program that enhanced end-of-life care for patients who are dying.

## Abbreviations

BC: British Columbia
CME: Continuing Medical Education
EOL: End of Life
GP: General Practitioner
HCC: Home and Community Care
MOA: Medical Office Assistant
PC: Palliative Care
PSP: Practice Support Program

## Footnotes

1 There were also a small number of questions about self-rated knowledge about the palliative approach to care, EOL resources in the community, billing, assessment tools that were introduced in the EOL module (viz., the Edmonton Symptom Assessment Scale (ESAS), the Palliative Performance Scale (PPS), and Seniors Assessment Tool (SAT). We do not, however, report on these in here, though we note that the GPs’ responses on these were similarly positive to those of the other questions.

2 We examined whether the adopter groups differed on any of the seven socio-demographic variables. All p-values were greater than .103, except for type of community, *χ*^2^(12) = 118.806, p < .001. The majority of early adopters were predominantly from urban/suburban communities (71.7 %) whereas the majorities of middle (68.4 %) and late (52.6 %) adopters were from metropolitan areas.

3 Years in practice was a continuous variable and its relationship with the scale scores was assessed with bivariate Pearson product correlation coefficients.

4 Type of adopter was not included in these analyses as an independent variable because the group definitions differed across the three survey points in time, and in particular, there were no “late” adopters on the follow-up survey, which was conducted about a year after the beginning of the module launch, and these GPs would not have completed the EOL training by that point.
